# Structure, Immunoreactivity, and In Silico Epitope Determination of SmSPI *S. mansoni* Serpin for Immunodiagnostic Application

**DOI:** 10.3390/vaccines9040322

**Published:** 2021-04-01

**Authors:** Stefano De Benedetti, Flavio Di Pisa, Enrico Mario Alessandro Fassi, Marina Cretich, Angelo Musicò, Roberto Frigerio, Alessandro Mussida, Mauro Bombaci, Renata Grifantini, Giorgio Colombo, Martino Bolognesi, Romualdo Grande, Nadia Zanchetta, Maria Rita Gismondo, Davide Mileto, Alessandro Mancon, Louise Jane Gourlay

**Affiliations:** 1 Department of Biosciences, Università degli Studi di Milano, Via Celoria 26, 20133 Milano, Italy; stefano.debenedetti@unimi.it (S.D.B.); DiPisa.Flavio@hsr.it (F.D.P.); martino.bolognesi@unimi.it (M.B.); 2 Consiglio Nazionale delle Ricerche, Istituto di Scienze e Tecnologie Chimiche “Giulio Natta” (SCITEC), Via Mario Bianco 9, 20131 Milano, Italy; enrico.fassi@unimi.it (E.M.A.F.); marina.cretich@cnr.it (M.C.); angelo.musico94@gmail.com (A.M.); roberto.frigerio94@gmail.com (R.F.); alessandro.mussida@scitec.cnr.it (A.M.); 3 Dipartimento di Scienze Farmaceutiche, Università degli Studi di Milano, Via L. Mangiagalli 25, 20133 Milano, Italy; 4 Istituto Nazionale Genetica Molecolare, Padiglione Romeo ed Enrica Invernizzi, IRCCS Ospedale Maggiore Policlinico, 20122 Milan, Italy; bombaci@ingm.org (M.B.); grifantini@ingm.org (R.G.); 5 Dipartimento di Chimica, Università di Pavia, V.le Taramelli 12, 27100 Pavia, Italy; g.colombo@unipv.it; 6 Centro di Ricerca Pediatrica Romeo ed Enrica Invernizzi, Università degli Studi di Milano, 20133 Milano, Italy; 7 UOC Microbiologia Clinica, Virologia e Diagnostica delle Bioemergenze ASST FBF Sacco, 20157 Milano, Italy; grande.romualdo@asst-fbf-sacco.it (R.G.); nadia.zanchetta@asst-fbf-sacco.it (N.Z.); mariarita.gismondo@unimi.it (M.R.G.); davide.mileto@unimi.it (D.M.); alessandro.mancon@unimi.it (A.M.); 8 Clinical Microbiology, Virology and Bioemergency Unit, Department of Biomedical and Clinical Sciences, Luigi Sacco Hospital, University of Milan, 20157 Milan, Italy

**Keywords:** Neglected Tropical Disease, circulating antigen, crystal structure, in silico epitope predictions, schistosomiasis, Serine protease inhibitor, immunodiagnostics

## Abstract

The human parasitic disease Schistosomiasis is caused by the *Schistosoma* trematode flatworm that infects freshwaters in tropical regions of the world, particularly in Sub-Saharan Africa, South America, and the Far-East. It has also been observed as an emerging disease in Europe, due to increased immigration. In addition to improved therapeutic strategies, it is imperative to develop novel, rapid, and sensitive diagnostic tests that can detect the *Schistosoma* parasite, allowing timely treatment. Present diagnosis is difficult and involves microscopy-based detection of Schistosoma eggs in the feces. In this context, we present the 3.22 Å resolution crystal structure of the circulating antigen Serine protease inhibitor from *S. mansoni* (SmSPI), and we describe it as a potential serodiagnostic marker. Moreover, we identify three potential immunoreactive epitopes using in silico-based epitope mapping methods. Here, we confirm effective immune sera reactivity of the recombinant antigen, suggesting the further investigation of the protein and/or its predicted epitopes as serodiagnostic Schistosomiasis biomarkers.

## 1. Introduction

*Schistosoma mansoni* is a helminth parasite etiological for the human disease known as Bilharzia or schistosomiasis. Schistosomiasis is a so-called Neglected Tropical Disease (NTD) that affects 230 million people with 700 million individuals at risk, mostly in Africa and the Middle East (*S. mansoni, S. intercalatum* and *S. haematobium*), but also in South America (*S. mansoni*), China, and the Philippines (*S. japonicum, S. mekongi*) [1,2]. Due to climate change and increased immigration, Schistosomiasis is also an emerging disease in Europe [3].

The Schistosoma pathogen is a trematode, parasitic flatworm, whose lifecycle begins in freshwaters, where its intermediate host, a snail belonging to *Biomphalaria bulinus* and *oncomelania* species, resides. The cercarial form of the parasite attaches to and penetrates the skin of the human host that is in contact with in infected waters [1]. Upon host entry, the cercarial form develops into the adult parasite. Females lay eggs that are moved towards the intestine and excreted in the feces and urines, depending on the *Schistosoma* species. The clinical outcomes present due to eggs that are not excreted, which become permanently lodged in the liver and intestine (*S. mansoni, S. japonicum, S. mekongi,* and *S. intercalatum*) or in the urogenital system and bladder (*S. haematobium*), where they can induce a granulomatous host immune response, resulting in chronic inflammation and disease manifestation. Acute symptoms are non-specific and can occur months after infection [4]. Symptoms present, even after decades, with severe intestinal and urogenital disorders [1].

Currently, the only safe and effective drug available to cure schistosomiasis is the chemotherapeutic agent Praziquantel. Despite its unknown specific mechanism of action, it has also been used as a prophylactic agent [5]. However, it is a great risk to base the treatment on a single drug, especially since susceptibility to praziquantel can vary amongst the same *Schistosoma* species, is dependent on the parasite lifecycle stage, and potential emerging drug resistance has been reported [6,7].

In addition to evaluate alternative schistosomiasis therapies, a rapid and highly sensitive diagnostic test that can detect asymptomatic *Schistosoma* infections is essential to control further diffusion of this parasite. Current diagnostic “gold standard” schistosomiasis tests detect the parasite’s eggs in urine and feces; however, poor test sensitivity cannot accurately rule out parasite presence [8]. Although DNA detection in excreta allows greater sensitivity, sampling is difficult, due to the uneven distribution of eggs in specimens [9]. The detection of the Cathode Circulating Antigen (Rapid Medical Diagnostics, Pretoria, South Africa) seems to be more effective than stool examination for the diagnosis in endemic countries, but it showed poor sensitivity in non-endemic regions [1,10]. While the specificity of current tests searching for circulating antibodies is often close to 100%, sensitivity is still sub-optimal. The newest and most promising developed diagnostic tools all involve patient’s antibody recognition of schistosome antigens using different approaches: Western blot, Immune-Chromatographic Test, and ELISA [10].

In this context, reverse and structural vaccinology methods can aid both antigen identification and the engineering of epitope-based biomarkers. 3D structure-based in silico prediction of immunoreactive epitopes on protein antigens from different pathogens has been shown to effectively lead to the development of diagnostic immunoreagents that may be presented in specific orientations on chemical scaffolds [11,12]. Currently, several highly accurate in silico tools exist that able to predict linear epitopes based on the antigen sequence. However, thanks to machine learning evolution, also conformational epitopes can be nowadays analyzed and predicted with accuracy [13,14,15]. Once identified, immunoreactive peptides can be chemically synthesized, immobilized, and oriented on a surface to create immunodiagnostic microarray platforms. This approach allows the simultaneous detection of different target molecules in a single experiment, where the epitopes act as a bait able to capture antibodies from a biological sample of an infected subject [16]. Furthermore, the selection of appropriate immunoreactive epitopes, besides detecting the presence of an infection, can also enable the discrimination between different antibody classes [17], giving information on the stage of infection and the disease progression. Finally, the multiplexing of different probes from different antigens can enhance the diagnostic potential of a single kit by allowing the simultaneous discrimination of different pathogens, offering fast and accurate diagnosis in a simple setting, optimal for on field large scale examination of patients in low resource countries [11]. 

The crucial and mandatory step that enables the pursuing of this innovative approach is the knowledge of the three-dimensional structure of the antigen of interest. Circulating antigen detection is currently attracting more and more attention, since it has been proven valuable for population screening and effective in correlating active infection and effects of treatment [18].

This study focuses on the circulating antigen Serine protease inhibitor (Serpin family) from *S. mansoni* (SmSPI). Serpins from parasites are interesting immunodiagnostic targets, due to their presence at the parasite-host interface, where they are crucial for the adaptation and survival of the parasite to the host environment by modulating inflammatory response [19]. Serpins are proteins distributed among all kingdoms of life that act mostly as Serine protease inhibitors, but also as inhibitors of other classes of proteases, although some that lack inhibitory activity can serve as chaperones or storage proteins [20]. Several Serpins have been identified in *S. mansoni*, highlighting the functional relevance of this class of protease inhibitors. Among these, SmSPI is able to weakly inhibit chymotrypsin and is expressed mostly in the parasite’s head gland [21], confirming its localization in the parasite-host interface, thus its potential as a diagnostic target.

In a previous study, SmSPI was immunorecognized by plasma antibodies from patients infected with *S. mansoni*, with higher sensitivity and comparable specificity to the gold standard [22]. Furthermore, the immune reactivity of SmSPI was demonstrated to be species-specific, and antibodies raised against its *S. haematobium* homolog (76% sequence identity) did not recognize SmSPI. Interestingly, the opposite is not true, as *S. haematobium* Serpin showed cross reactivity against plasma antibodies from *S. mansoni*-infected patients, although reactivity was inferior with respect *to S. haematobium* antibodies [22]. This suggests that despite their high sequence identity, reactive epitopes are not entirely conserved, or/and they depend on 3D conformation. The determination of SmSPI 3D structure could point out such structural differences. Furthermore, the in silico discovery of immunoreactive epitopes may allow the design of species-specific epitopes for immunodiagnostic purposes.

In this context, we present the 3.22 Å resolution crystal structure of the *S. mansoni* circulating antigen SmSPI together with flanking immune sera recognition tests using sera from affected patients. Finally, starting from the crystal structure, we report the in silico prediction of its immunoreactive epitopes for further evaluation as diagnostic biomarkers for schistosomiasis detection. 

## 2. Materials and Methods

### 2.1. Cloning, Overexpression, and Purification

The Smp_090080 gene sequence, coding for residues 20-402 (corresponding to the Serpin domain) of the protein SmSPI (Uniprot code G4LYU6), was purchased as a synthetic gene (Biomatik) and cloned into the pTXB1 vector (New England Biolabs, Ipswich, United States) via *NdeI* and *SapI* restriction sites, for bacterial expression with the C-terminal fusion protein Mxe GyrA intein and chitin-binding domain (CBD) for tag-free protein purification. Overexpression was performed in Luria Bertani LB medium overnight at 20 °C in BL21 (DE3) pLysS cells after induction with 0.4 mM isopropyl β-d-1-thiogalactopyranoside. Cells were harvested at 6500 rpm at 4 °C, resuspended in the column loading buffer (20 mM Tris-HCl pH 8.5, 500 mM NaCl), and lysed at a constant pressure (25,000 lb/in^2^) with a cell disruptor (Constant Systems Ltd., Daventry, United Kingdom). Affinity purification was performed in a gravity flow setup in Econo-Column^®^ Chromatography Columns, 1.0 cm × 10 cm (Bio-rad, Hercules, California, United States), packed with 2 mL chitin resin (New England Biolabs) equilibrated with column loading buffer. Lysed supernatant was centrifuged 16,000 rpm at 4 °C for 20 min, filtered with 0.22 µm pore size Millex^®^ syringe filters (Merck, Darmstadt, Germany) and slowly loaded onto the column. Impurities were washed with 50 column volumes of wash buffer (20 mM Tris-HCl pH 8.5, 1 M NaCl). The tag-free protein was eluted after overnight incubation at 4 °C with 50 mM DTT that induces the intein-mediated self-cleavage of the CBD tag and concentrated with Amicon^®^ Ultra 15 mL Centrifugal Filters (Merck). A final purification step by size exclusion chromatography on a Superdex 75 16/60 prep grade column (GE Healthcare, Chicago, Illinois, United States) was carried out in 10 mM Tris-HCl pH 8.5, 50 mM NaCl, 10% (*v*/*v*) glycerol, 2 mM DTT. All chromatographic procedures were performed at room temperature.

### 2.2. Crystallization

SmSPI crystals were grown using the vapor diffusion method, set-up in sitting drops using the Orxy4 crystallization robot (Douglas Instruments, Hungerford, United Kingdom). Briefly, SmSPI (10.5 mg/mL) was deposited at 3 different concentrations (30%, 50%, 70%) in 400 nL drops, in flat CrystalQuick 96-well sitting drop plates (Greiner Bio-One, Kremsmünster, Upper Austria). Each reservoir contained 100 µL of 96 different crystallization conditions from the Morpheus screen (Molecular Dimensions, Newmarket, Suffolk, United Kingdom). Tiny, needle-shaped crystals grew after 3 weeks at room temperature in a drop containing 70% protein in conditions 2–5 (10% (*w*/*v*) PEG 20,000, 20% (*v*/*v*) PEG MME 550, 0.03 M each of diethyleneglycol, triethyleneglycol, tetraethyleneglycol, pentaethyleneglycol in buffer 0.1 M MOPS/HEPES-Na pH 7.5). Crystals were directly cryocooled in liquid nitrogen due to the cryoprotective properties of the crystallization solution. 

### 2.3. X-ray Diffraction Data Collection

X-ray diffraction data were collected at a resolution of 3.22 Å on a single crystal on the I24 beamline at the Diamond Light Source (DLS—Harwell Science and Innovation Campus) using a Pilatus3 X 6M detector. This beamline was chosen because it is equipped with a tunable microfocus, set at 7 × 7 µm, optimal for the reduced crystal size. Data were processed with XIA2/DIALS and assigned to the spacegroup P 3_2_ 2 1 with a unit cell of a = b = 98.9, c = 115.3 Å, α = β = 90°, γ = 120° [23]. The structure was solved by molecular replacement using MOLREP [24] from the CCP4i2 suite, and the structural homolog from *Schistosoma haematobium* (PDB: 3STO [25]) with whom it shares 76% sequence identity, as a search model. The model was further manually inspected and built using Coot [26] and refined using PHENIX.refine [27]. The final model was then validated with MolProbity [28]. Atomic coordinates and structure factors were deposited in the PDB (www.rcsb.org, accessed on 30 September 2020) under accession code 6SSV.

### 2.4. Sample Collection

Twelve sera from Schistosome patients (SM) were collected by Sacco Hospital in Milan. Ten sera belonged to subjects originating from Egypt, one to an Italian subject and one patient originated from Sub Saharan Africa. All subjects were positive for Schistosome IgG (Bordier Affinity Products CH). Among these, parasite eggs were detected in only two subjects: one had eggs in feces and one in the urine. The ethical committee of Sacco Hospital in Milan approved the use of sera specimens for research purposes and informed consent was obtained from the subjects involved in the study. Healthy Donor sera (HD), matched for sex and age, were obtained from the institute’s in-house biobanks.

### 2.5. Dissociation-Enhanced Lanthanide Fluoroscence ImmunoAssays (DELFIA)

The reactivity of SmSPI recombinant protein with human sera was confirmed by DELFIA^®^ assay, a time-resolved fluorescence method, used as described previously [29]. In brief, 20 μg/mL purified recombinant SmSPI were coated in a 384-well format plates in quadruplicate. The procedure of serum assay was automatically performed by Freedom-EVO Liquid Handling (Tecan, Männedorf, Switzerland). Plates were then blocked for 1 h at 37 °C with a blocking reagent (PBS, 2% (*w*/*v*) bovine serum albumin (BSA)). Serum samples, were diluted 1:300 in 0.1 % (*v*/*v*) TPBS with 1% (*w*/*v*) BSA and incubated for 1 h at 37 °C. After washing (5 times) with washing buffer (PerkinElmer, Waltham, Massachusetts, United States) plates were incubated for 30 min at room temperature in the dark with Europium-labeled α-Human IgG serum (1:500 in diluting buffer, PerkinElmer). After extensive washing by Hydrospeed™ (Tecan), plates were left at RT for 10 min and then read on an Infinite F200 PRO instrument (Tecan). Fluorescence intensity values higher than the mean of Healthy Donors (HD) plus one standard deviation were considered as positive. DELFIA results were analyzed using the two-tailed *X*^2^ test, and the Student’s t test, using GraphPad software. Sensitivity was defined as the true positive rate in %, while specificity was defined as the true negative rate in Healthy Donor (HD) subjects in %.

### 2.6. Protein Microarrays

Silicon slides were coated by MCP2 (Lucidant Polymers, Sunnyvale, United States), as previously described [30,31]. SmSPI was dissolved in PBS to 1 mg/mL using a non-contact Spotter S12 (Scienion Co., Berlin, Germany). Printed slides were placed in a humid chamber overnight at room temperature. Slides were then blocked with Blocking solution (50 mM ethanolamine dissolved in water, pH 9) for 1 h, washed with distilled water, and dried under a nitrogen stream.

All samples were then diluted 1:50 in the incubation buffer (50 mM Tris/HCl pH 7.6, 150 mM NaCl, 0.02% (*v*/*v*) TWEEN 20) with 1% (*w*/*v*) BSA and incubated dynamically for 1 h at room temperature. Slides were washed 3 times for 1 min with washing buffer (50 mM Tris/HCl pH 9, 250 mM NaCl, 0.05% TWEEN 20). The second incubation with secondary antibody was performed with Anti-Human IgG-Cy3 (Jackson ImmunoReserarch, West Grove, United States) diluted 1:1000 in incubation buffer 1% (*w*/*v*) BSA. Finally, slides were washed and dried and the analysis were performed by TECAN power scanner at 50% laser intensity and 100% gain. In total, 28 samples were tested (14 healthy serums and 12 samples affected by Schistosomiasis). Sensitivity and specificity were calculated, as previously described.

### 2.7. Molecular Dynamics Simulations

The MD simulation package Amber 16 was used to perform computer simulations on the crystal structure of SmSPI using the Amber-ff14SB force field [32]. The system was fully solvated in a TIP3P [33] water box, counter ions were added to neutralize the system and ensure overall charge neutrality, and octahedral cubic periodic boundary conditions were imposed in the three dimensions. After minimization, the system was subjected to an equilibration phase where temperature was slowly increased to 300 K in 300 ps, and pressure was increased to 1 atm. Water molecules and protein heavy atoms were position restrained. Next, three independent replicas of 1 μs each (3 μs in total) of unrestrained simulations were run in an NPT ensemble; a Langevin equilibration scheme thermostat [34] and Berendsen thermostat Monte Carlo barostat [35] were used to keep constant temperature and pressure (1 atm), respectively. MD simulations were run using the PMEMD code in the GPU accelerated version [36] with a time step of 2 fs. Electrostatic forces interactions were evaluated by using the Particle Mesh Ewald method and Lennard-Jones forces and setting a cut-off of 9 Å. All the bonds involving hydrogen atoms were constrained using the SHAKE algorithm [37]. MD analyses were carried out on a meta-trajectory (heavy atoms), obtained by concatenating all three independent trajectories. To define the structure on which to apply the MLCE epitope prediction, the Daura clustering algorithm was used [38]. 

### 2.8. Prediction of Epitopes: MLCE

Epitope predictions were carried out using the Matrix of Local Coupling Energies (MLCE) method [39], which combines the energetic profile of a given protein with the analysis of its structural/dynamical determinants. MLCE permits to determine non-optimized/low intensity energetic interaction-networks, which corresponds to those regions of the protein that can be more prone to establish interactions with antibodies. This method has been widely validated and described elsewhere [40,41,42], see Supporting Information for details. Briefly, the contiguous regions on the protein surface which are deemed to have minimal coupling energies with the rest of the structure are selected on the basis of the eigenvalue decomposition of the matrix reporting the non-bonded interaction of all residue-pairs. Filtering of the simplified matrix with the contact matrix allows the identification and selection of residue pairs which show minimal coupling with the rest of the protein structure (i.e., epitopes). Selection is carried out on the basis of a threshold value (called softness), which defines the percentage of the set of putative interaction sites by including the increasing residue-residue coupling values until the number of couplings that correspond to the lowest contact-filtered pairs under the threshold was reached. In this work, MLCE analysis was performed using the most restrictive level (5%) of prediction softness.

## 3. Results and Discussion

### 3.1. SmSPI Expression and Purification

SmSPI was recombinantly expressed in BL21 (DE3) pLysS *E. coli* cells as a fusion protein, with a Mxe GyrA intein and chitin-binding domain (CBD) at its C-terminal end, as described in Materials and Methods. An Alanine residue was added between SmSPI and the fusion protein to enhance the self-cleavage of the tag. SmSPI was expressed in soluble form and in SDS-PAGE experiments, which were run with an apparent molecular weight (MW) of 44 kDa, in agreement with the calculated MW after the cleavage of the tag, on a 12% Tris-glycine precast gel (Genscript) run in 1x MOPS (Figure S1). Protein yields were 1.5 mg per liter of bacterial culture.

### 3.2. SmSPI Crystal Structure

**Figure 1:**
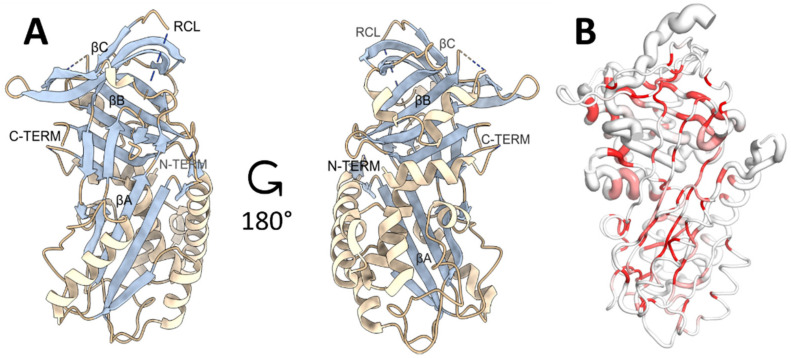
The crystal structure of Serine protease inhibitor from *S. mansoni* (SmSPI) (PDB 6SSV). (**A**) Cartoon representation of the secondary structure organization: alpha helices (bisque) and beta sheets (light blue) are highlighted and named after conventional Serpin nomenclature. Gaps in the structure are indicated by dashed lines. (**B**) Sausage representation of the conserved regions among 153 structural homologs: the size of the tube is proportional to the mean r.m.s. deviation per residue between Cα pairs. White: low sequence conservation; Red: sequence identity. Graphic representations were generated with UCSF Chimera software (Version 1.13.1, The Regents of the University of California, Oakland, CA, USA) and The PyMOL Molecular Graphics System, Version 2.0 Schrödinger, LLC, New York, NY, USA [47,48].

One protein molecule was found in the crystal asymmetric unit, with an estimated solvent content of 67 % (Matthew’s coefficient = 3.78 Å^3^/Da) (Figure 1). Parameters for data collection and refinement are reported in Table S1.

The SmSPI structure presents the fold and topology of a canonical Serpin composed of 3 β-sheets (named βA, βB, βC, assigned according to the conventional Serpin nomenclature [43]) and 10 α-helices (Figure 1A). Electron density was well defined for the main chain atoms except for some flexible, loop regions. The major region with no electron density is the Reactive Centre Loop (RCL; residues 357 to 368), which is highly flexible and solvent exposed; its function is indeed to act as a bait for the target protease to exert the Serpin inhibitory activity. Following the cleavage of the RCL, the target protease remains covalently bound to the Serpin and is translocated on the other side of the protein inhibitory molecule through the insertion of part of the RCL in the β-sheet A. This leads to a massive distortion of the active site of the target protease that is thus inactivated [44]. Other poor electron density regions were for several solvent exposed loops (AA 35–36, 107–108, 262–263), in accordance with other deposited Serpin structures. 

3D structure-based comparison performed with the DALI server (http://ekhidna2.biocenter.helsinki.fi/dali/; accessed on 9 January 2020 [45]) revealed, as expected, that the closest structural homologue (RMSD for Calpha atoms of 1.1 Å) is ShSPI, the Serpin from *S. haematobium* (sequence identity of 76%). Superimposition of SmSPI with all known homologous proteins in the PDB was carried out using the ENDscript (http://endscript.ibcp.fr/ESPript/ENDscript/ accessed on 9 January 2020 [46]) (Figure 1B). The differences in the main chain atoms between SmSPI and the 153 structural homologs analyzed by the software (Figure S2) reside in the RCL region and its following βB sheet, as highlighted in Figure 1B by the thick tube corresponding to RCL. This can be expected, since the RCL gives the specificity of the target to each Serpin and indeed is the most flexible and exposed region in each member of this inhibitory family as well as the most variable in the amino acid sequence.

### 3.3. Immune Sera Reactivity Tests

Twelve patients (8 male and 4 female) between 38 and 61 years of age, living in the Lombardy region of Italy, infected by *Schistosoma* (as revealed by IgG analysis on Soluble Egg Antigens) were recruited for the study. The purified recombinant protein was tested for its ability to detect serum antibodies. The immunoreactivity efficacy of the recombinant protein was tested with two different, approaches. Both confirmed SmSPI as a valid target for further development for serological-based immunodiagnostic tests.

#### 3.3.1. SmSPI Was Confirmed to Be Highly Associated to Patients Infected by *S. mansoni* and Was Able to Discriminate the Healthy Donor

**Figure 2:**
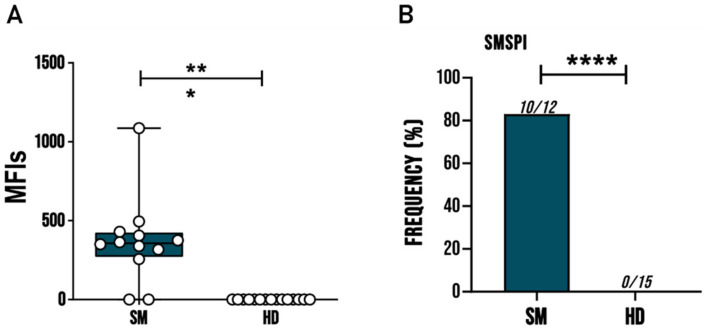
SmSPI recombinant protein highlights a higher immunoreactivity in sera patients affected by *S. mansoni* than in that of healthy donors (HDs). (**A**) Whisker plots comparing mean fluorescence intensity (MFI) of SmSPI recombinant protein tested with sera of 15 HDs and 12 Schistosome patients (SM). Each dot represents the MFI of a single patient. Asterisk denotes statistical significance (Student’s *t* test, *p* < 0.01). (**B**) Recognition frequency. Each serum was tested four times. Asterisk denotes statistical significance (Fisher exact test, *p* < 0.01). *** *p* < 0.001, **** *p* < 0.0001.

In order to assess the reactivity of sera from patients infected by *S. mansoni* on SmSPI recombinant protein, we used DELFIA assay to screen a set of sera comprising 12 *S. mansoni* patients and 15 HDs. In this way, we observed that *S. mansoni* patients’ sera showed a significantly higher reactivity than HD sera, both in terms of signal and in terms of frequency with a value equal to 83.3% (Figure 2).

#### 3.3.2. Microarray Assays

**Figure 3:**
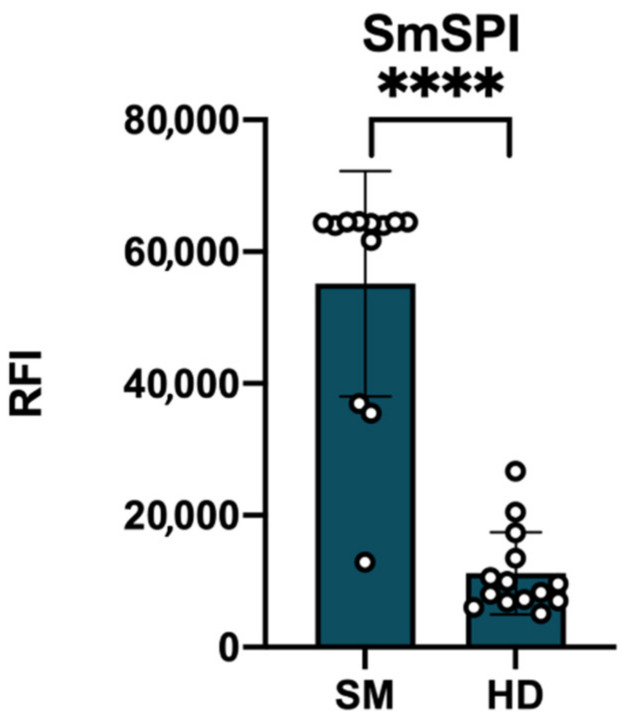
Unpaired *t*-Test results for the SmSPI specific human IgG. Protein arrays were probed with sera from Schistosome patients (N = 12) and control patients (N = 14). Significative: *p* < 0.05; **** *p* < 0.0001.

Slides spotted with SmSPI were probed with sera (N = 12) from Schistosome patients and healthy donors (N = 14). SmSPI showed to be able to bind human IgG in sera from people affected by Schistosomiasis, while the response of the protein to healthy serum resulted to be significantly lower (*p* < 0.001, Figure 3). Only one unhealthy subject showed no IgG signal against Serpin; the sensitivity of the assay was calculated to be 91.7%.

Sensitivity (defined as the true positive rate in %) of both tests resulted similar (83.3% and 91.7%), in line with previously reported results [22,49]. It is interesting to point out that only one sample from the schistosome patients (SM) group did not respond to Delphia assay, and the same provided RFI values comparable to the control group in the microarray assay. In light of these results, it may be either that this single subject was in a different stage of disease with respect to other patients or it is a confirmation of the unreliability of the current diagnostic tests especially in non-endemic regions [10]. Nevertheless, the selected antigen was shown to have the potential to be exploited as a target with a comparable diagnostic power to current standards. In light of this, we proceeded to further explore its structure in order to identify its immunoreactive epitopes with the aim to deepen its knowledge and increase its immunodiagnostic power.

### 3.4. In Silico Epitope Predictions

On the basis of the SmSPI crystal structure, in silico epitope predictions were performed to identify the most immunoreactive portion(s) of the protein that are likely to be recognized by patient antibodies. The identification of immunoreactive epitopes has the potential to lead to the development of synthetic peptides with improved immunological properties, that may further be implemented in a novel multiplexed immunodiagnostic platform [50]. 

Epitope mapping was carried out on the representative structure of SmSPI (that accounts for 90% of conformational ensembles explored) subjected to molecular dynamics simulations (three independent simulations of 1 µs each). Missing loops (G35-Q36, I107-D108, E262-K263) and RCL residues (from T357 to A368) were built by homology modelling using the SWISS-MODEL Workspace [51,52] web-tool. The structure of S. haematobium Serpin (PDB entry 3STO) was used as a template, due to its high structural similarity with SmSPI (76% sequence identity, RMSD for Backbone atoms of 1.03 Å).

**Figure 4:**
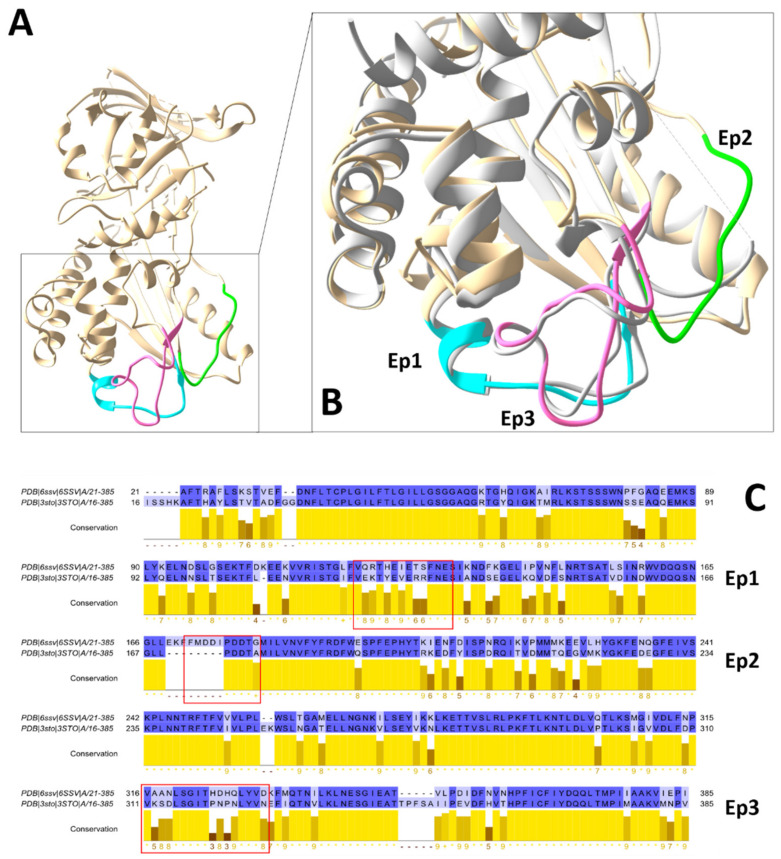
Representation of the three in silico identified epitopes, structure, and sequence comparison with S. hematobium homolog Serpin ShSPI. (**A**) SmSPI whole molecule representation. (**B**) SmSPI magnification of the three identified epitopes: Ep1 (cyan), Ep2 (green), and Ep3 (magenta) and structure comparison of the immunoreactive portion between SmSPI (6SSV—tan colored) and ShSPI (3STO—light grey colored). (**C**) Alignment of 6SSV and 3STO sequences. Percentage identity is color coded (darker blue: higher identity; lighter blue: lower identity). Conservation histograms of total alignment is reported in yellow. Alignment were performed with Jalview 2.11 [53].

Three SmSPI epitopes were identified: Ep1: 123-VQRTHEIETSFNE-135; Ep2: 175-FMDDIPDDTG-185; Ep3: 320-NPVAANLSGITHDHQLYVD-338 (Figure 4). The Root Mean Square Fluctuation (RMSF) was calculated on the protein backbone for all MD simulations, to ascertain whether predicted epitopes were effectively located in highly dynamic regions of the protein (i.e., more inclined to interact with an antibody). Our results show that all predicted epitopes correspond to RMSF peaks, in other words, portions of the protein that fluctuate and can aptly be engaged by antibodies in immune responses (Figure S3). 

All three identified epitopes are clustered together in the same region of the protein (Figure 4B). In particular, Ep1 and Ep3 potentially belong to the same conformational epitope. From a structural point of view, Ep1 almost perfectly overlaps with the equivalent region in S. haematobium Serpin (3STO), whereas Ep2 from SmSPI corresponds to a region that is not modelled in 3STO, probably due to high flexibility and thus lacks electron density, making any comparisons impossible (Figure 4B). Ep3 lies in one of the least conserved regions between the two proteins (Figure 4C), from both sequence and structural point-of-view. It is interesting to highlight that the Ep1 sequence, as well, is one of the least conserved among the whole protein sequence. These small sequence and structural differences could be at the basis of the species specific antibody response previously described [22]. Species-specific diagnosis is not presently required. Improving Schistosomiasis diagnosis overall is more pertinent, since Praziquantel is used to treat all species, however such differences may be interesting for the study of these antigens from a therapeutic, i.e., vaccine, point-of-view.

## 4. Conclusions 

In conclusion, this study describes a reliable workflow for the identification of immunoreactive epitopes that may be useful for the detection of schistosomiasis.

Starting from the knowledge that *Sm*SPI was immunorecognized by plasma antibodies from patients infected with *S. mansoni*, the crystal structure of the antigen was determined and in silico predictions of immunoreactive epitopes was carried out. Three major regions turned out to be good epitope candidates worthy of further characterizations.

Overall, our data have the potential to be further exploited for the rational design of immunodiagnostic kits aiming to a fast and reliable identification of the disease. Isolating only the epitope regions, allowing epitopes to be presented in optimal conformations, and multiplexing targets can improve the sensitivity of antibody detection.

## Data Availability

The data presented in this study are available on request from the corresponding author.

## Supplementary Materials

The following are available online at https://www.mdpi.com/article/10.3390/vaccines9040322/s1, Figure S1: SDS-PAGE and gel filtration chromatogram. Figure S2: Sequence alignments. Figure S3: Root Mean Square Fluctuation (RMSF) of SmSPI (3 × 1 μs MD simulations). Table S1: Data collection statistics and refinement parameters, Material and Methods: Detailed Prediction of epitopes: MLCE.
