# Supplementary material for: Structure, Immunoreactivity, and In Silico Epitope Determination of SmSPI S. mansoni Serpin for Immunodiagnostic Application
Source: Vaccines (Basel). 2021 Apr 1;9(4):322. doi: 10.3390/vaccines9040322 (PMC8066017; doi:10.3390/vaccines9040322)

## Slide 1
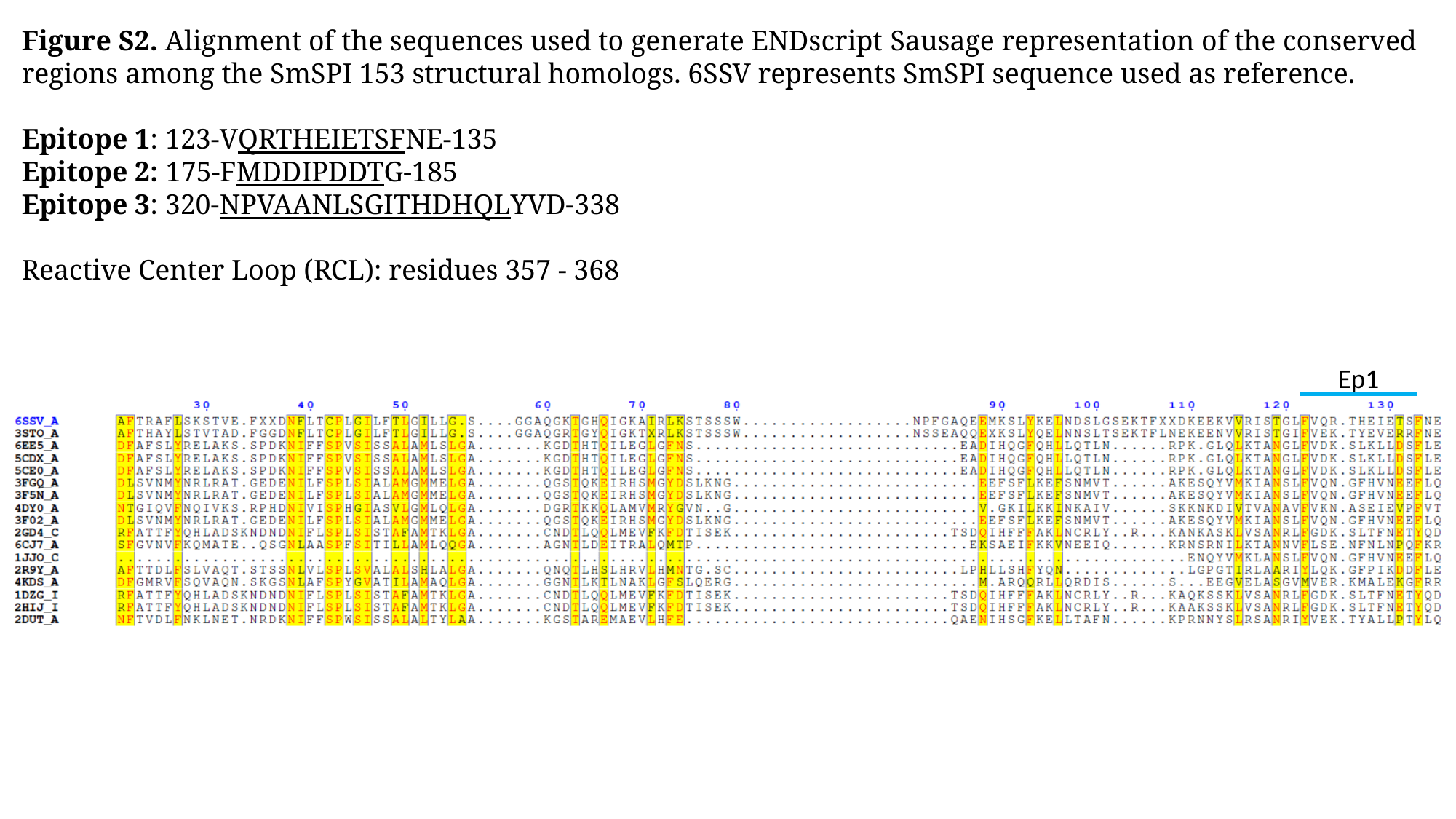

Figure S2. Alignment of the sequences used to generate ENDscript Sausage representation of the conserved regions among the SmSPI 153 structural homologs. 6SSV represents SmSPI sequence used as reference.
Epitope 1: 123-VQRTHEIETSFNE-135
Epitope 2: 175-FMDDIPDDTG-185
Epitope 3: 320-NPVAANLSGITHDHQLYVD-338
Reactive Center Loop (RCL): residues 357 - 368
Ep1

## Slide 2
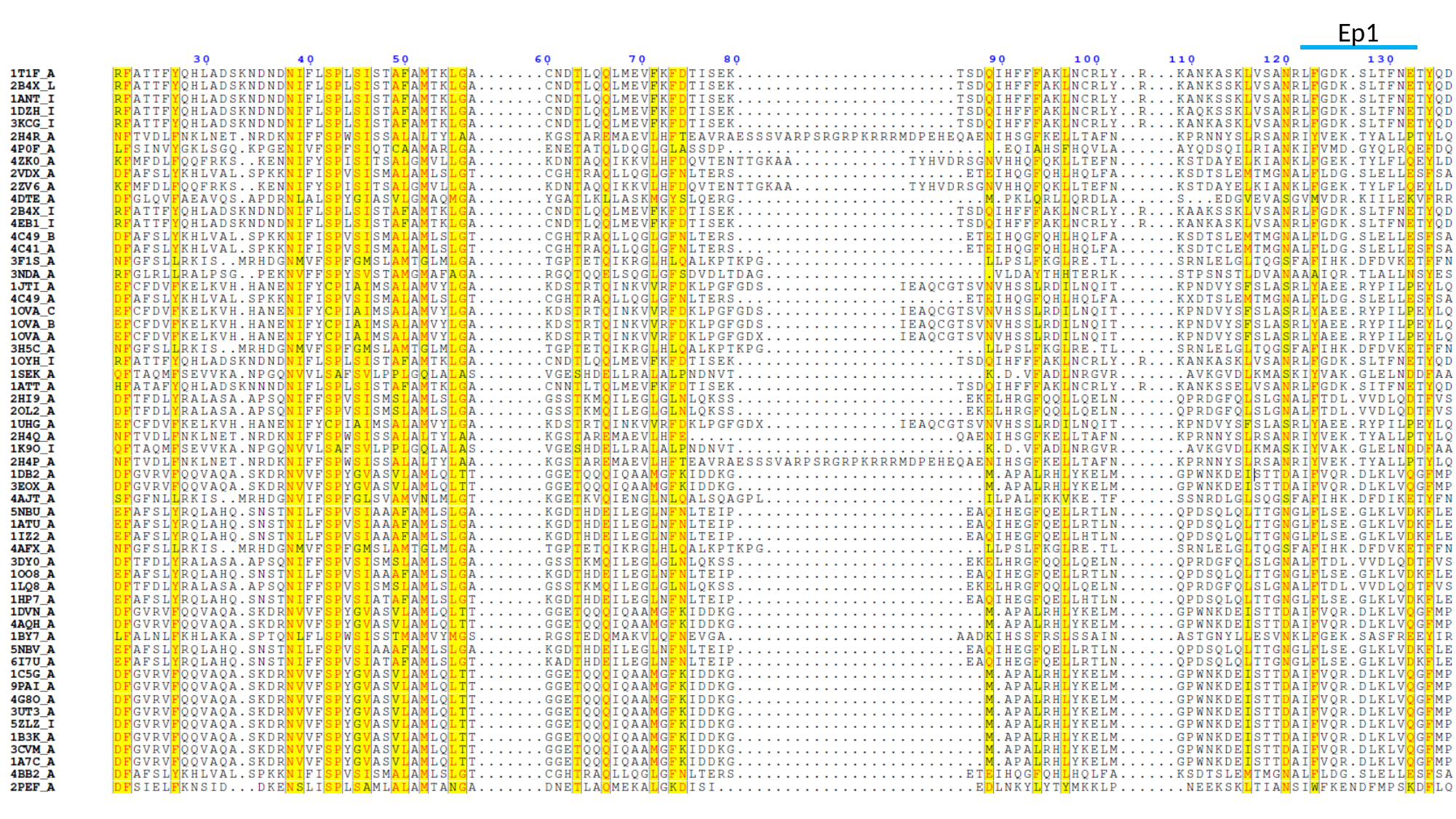

Ep1

## Slide 3
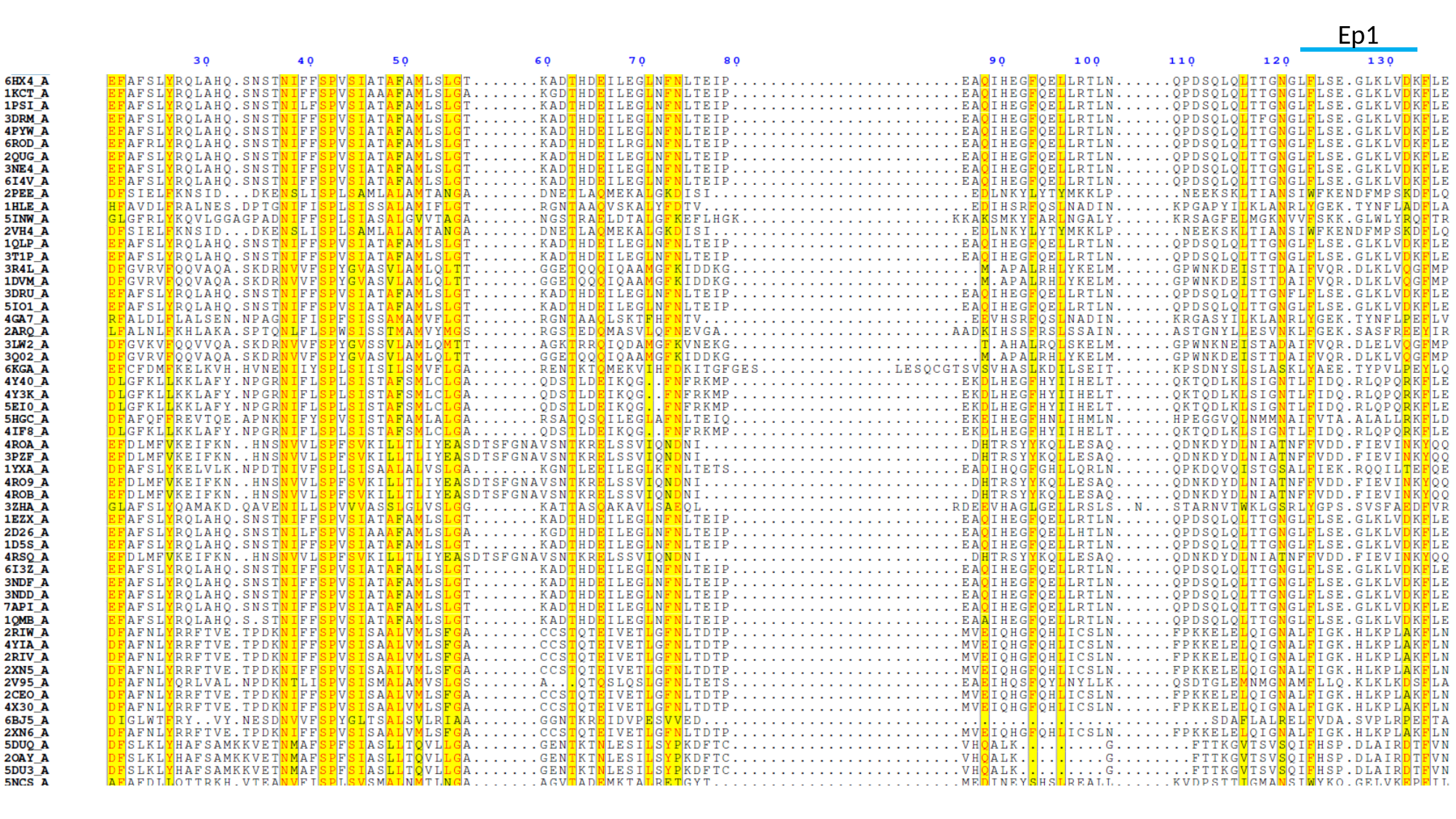

Ep1

## Slide 4
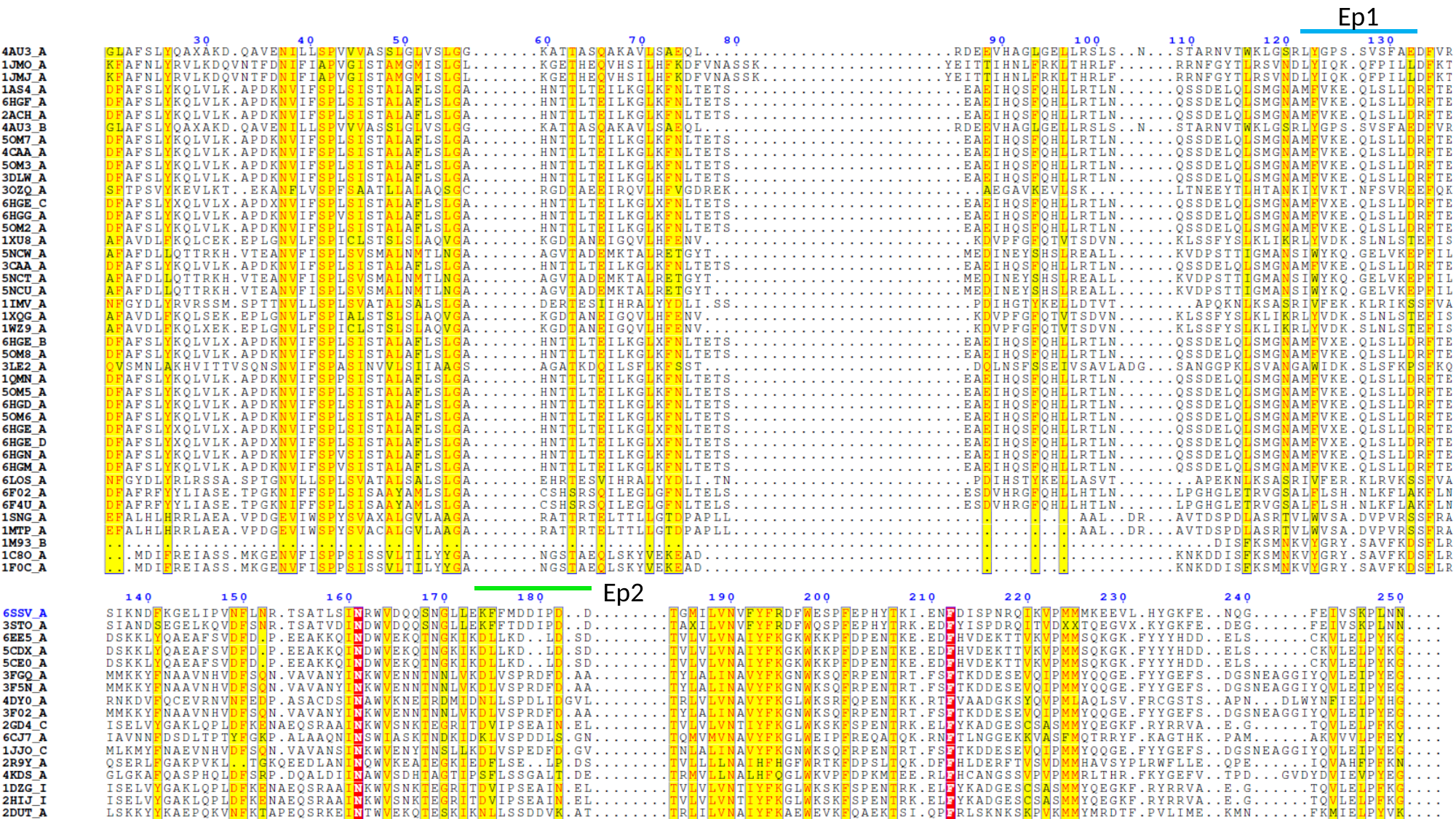

Ep1
Ep2

## Slide 5
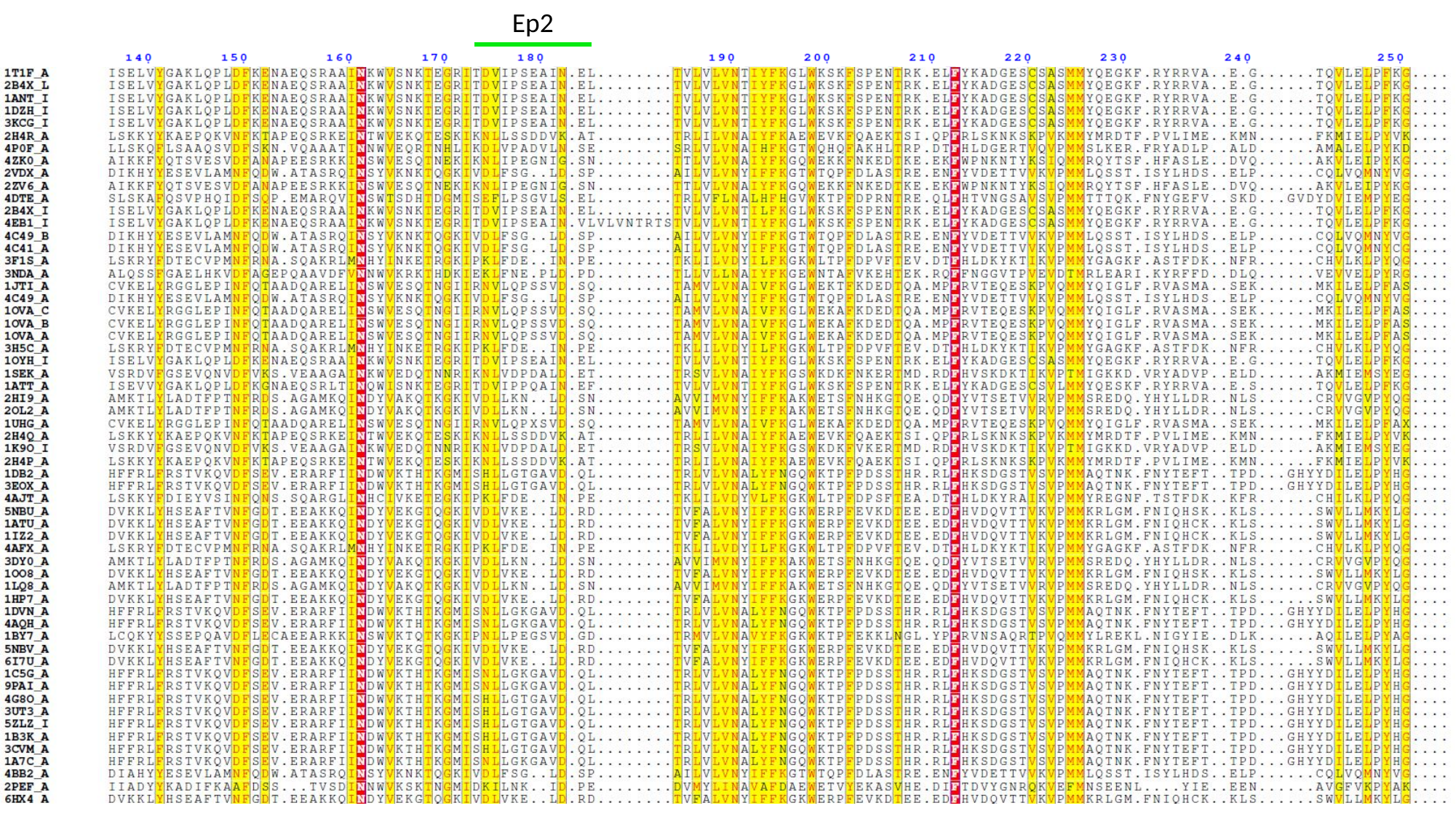

Ep2

## Slide 6
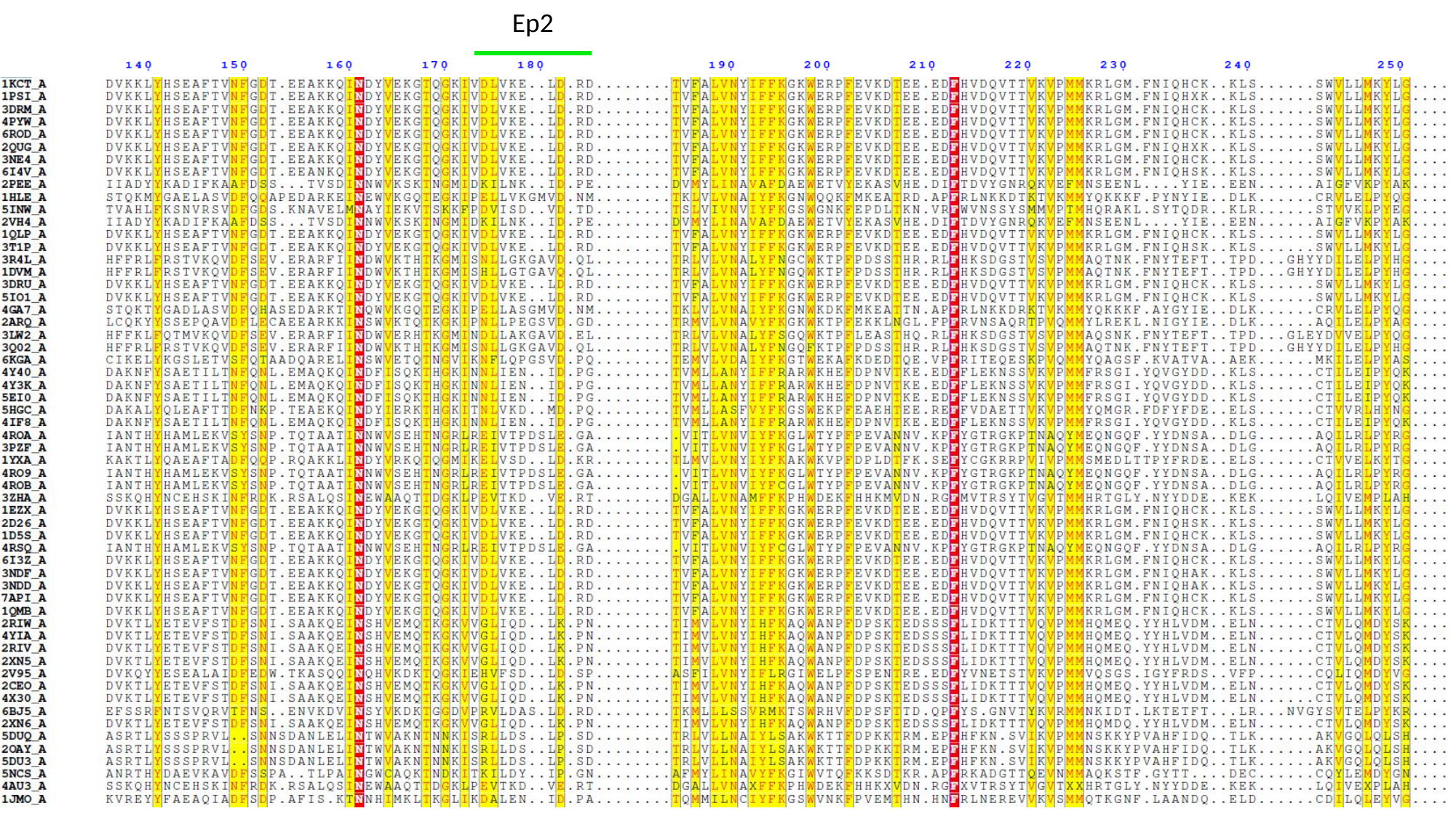

Ep2

## Slide 7
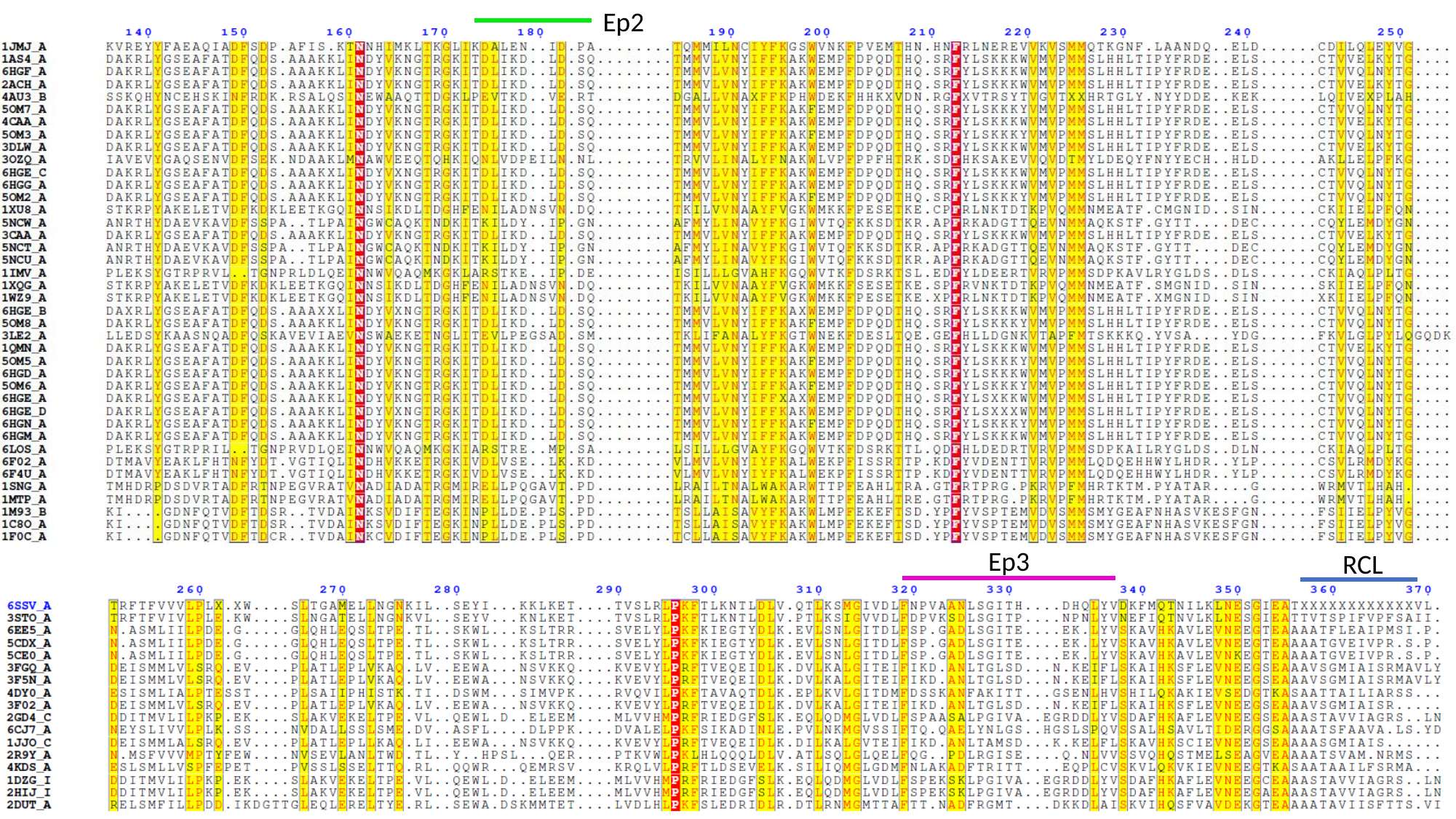

Ep2
Ep3
RCL

## Slide 8
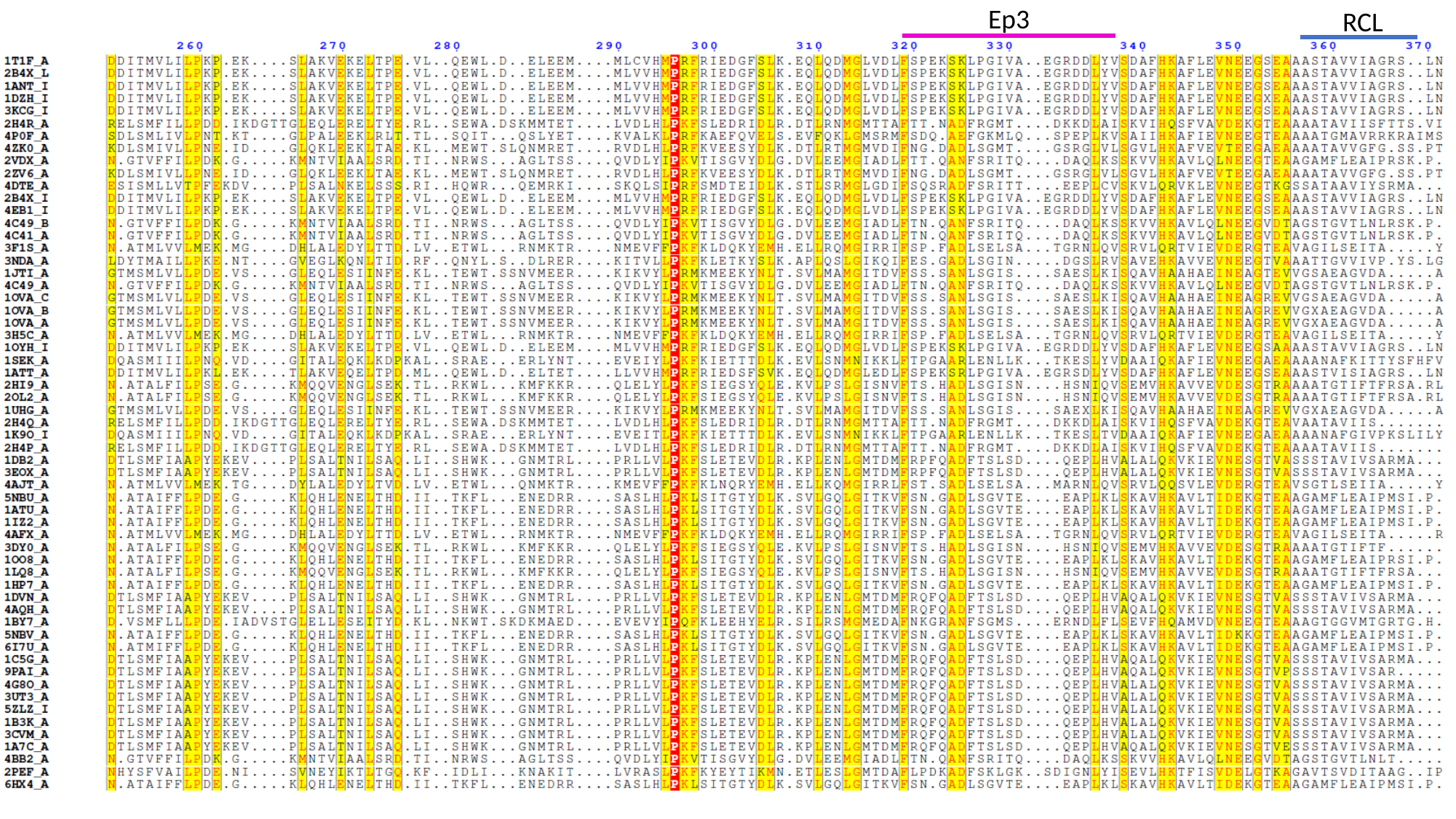

Ep3
RCL

## Slide 9
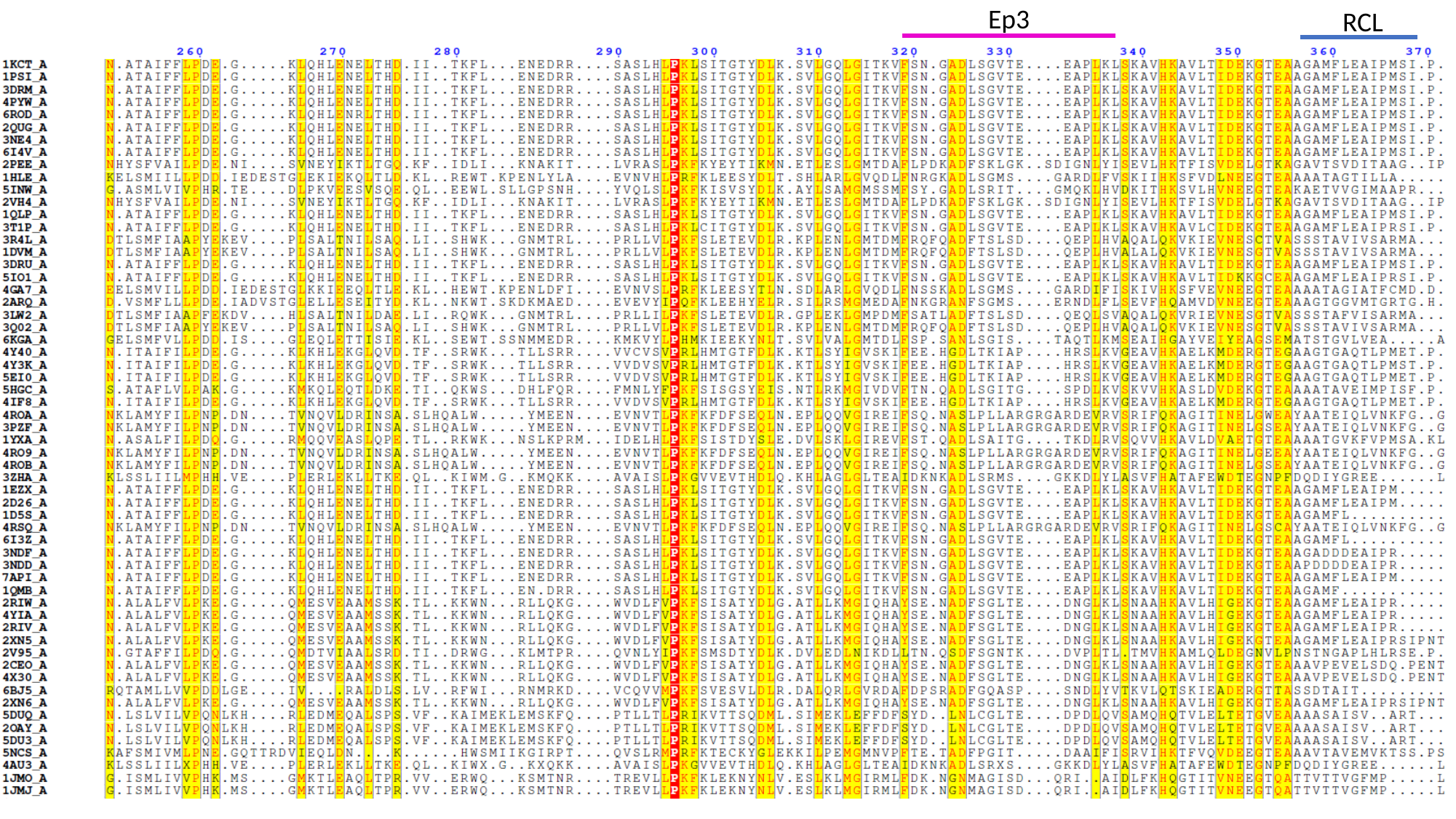

Ep3
RCL

## Slide 10
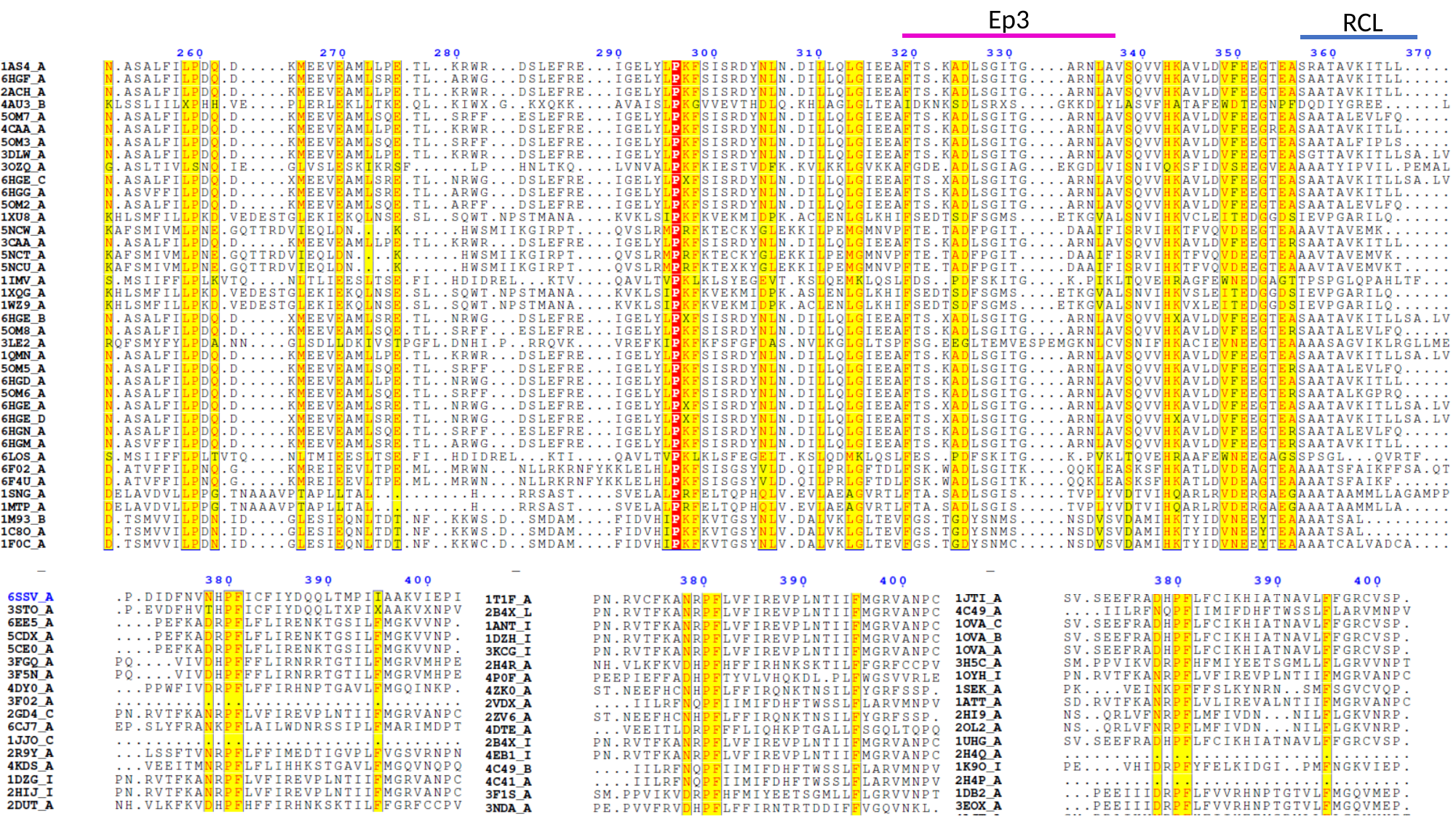

Ep3
RCL

## Slide 11
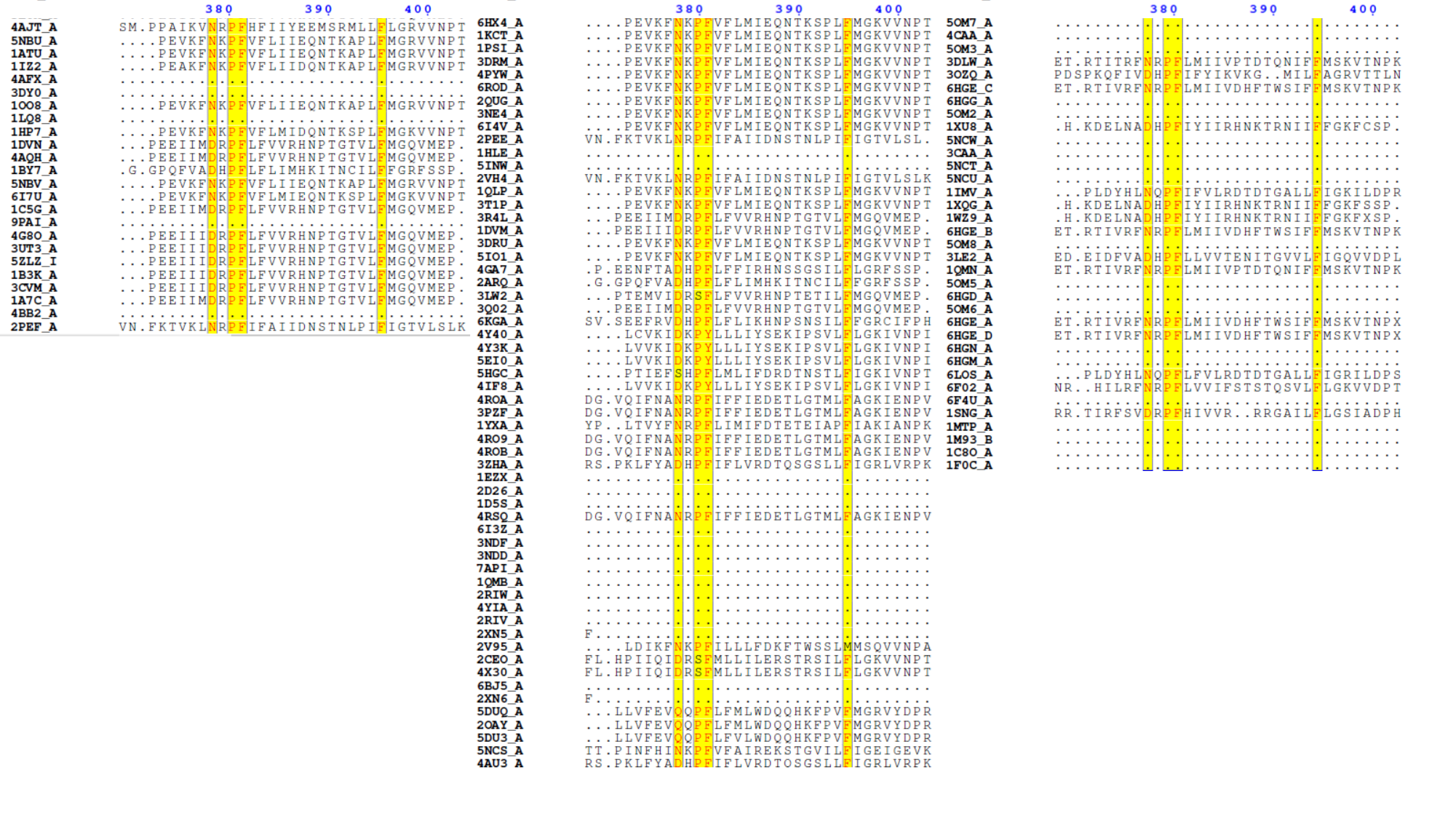

Supplement: Supplementary file 1 [file vaccines-09-00322-s001.zip › Figure S2.pptx]
