# Supplementary material for: Structure, Immunoreactivity, and In Silico Epitope Determination of SmSPI S. mansoni Serpin for Immunodiagnostic Application
Source: Vaccines (Basel). 2021 Apr 1;9(4):322. doi: 10.3390/vaccines9040322 (PMC8066017; doi:10.3390/vaccines9040322)
Supplement: Supplementary file 1 [file vaccines-09-00322-s001.zip › Manuscript_Vaccines_Supporting_Information_220221_FINALE_SUBMISSION.docx]

**SUPPORTING INFORMATION**

Structure, immunoreactivity and *in silico* epitope determination of SmSPI *S. mansoni* Serpin for immunodiagnostic application.

Stefano De Benedetti ^1§^, Flavio Di Pisa ^1Ψ^, Enrico M. A. Fassi ^2,3^, Marina Cretich ^2^, Angelo Musicò ^2^, Roberto Frigerio ^2^ , Alessandro Mussida ^2^, Mauro Bombaci ^4^, Renata Grifantini ^4^, Giorgio Colombo ^5^, Martino Bolognesi ^1,6^, Romualdo Grande ^7^, Nadia Zanchetta ^7^, Mariarita Gismondo ^7,8^, Davide Mileto ^7^, Alessandro Mancon ^7^, Louise Jane Gourlay *^1^.

**Table S1.** **Data collection statistics and refinement parameters.** SmSPI. X-ray diffraction data were collected on a single crystal. Parentheses indicate parameters related to the high-resolution shell 3.27 – 3.22 Å. Data were refined to 3.22 Å. ^a^*R*_merge_ =*R*_merge_ = ∑|I/ - <I>|/∑I x 100, where I is the intensity of a reflection and <I> is the average intensity. ^b^*R*_factor_ = Σ|Fo-Fc|/ΣFo x 100; ^c^For cross-validation, 5% experimental reflections were randomly selected to calculate the Rfree.

| Data collection | |
| --- | --- |
| Space group | P 3_2_ 2 1 |
| Cell dimensions | |
| *a*, *b*, *c* (Å) | 98.9, 98.9, 115.3 |
| α, β, γ (°) | 90, 90, 120 |
| Resolution (Å) | 3.22 (3.27 – 3.22) |
| *I* / σ*I* | 5.1 (0.9) |
| *CC _½_* | 0.966 (0.361) |
| *^a^R*_merge_ | 0.392 (3.064) |
| *R*_meas_ | 0.414 (3.234) |
| Completeness (%) | 99.8 (95.8) |
| Redundancy | 9.5 (9.8) |
| Refinement | |
| Resolution (Å) | 3.22 |
| No. unique reflections | 10967 |
| *^b^R*_factor_ / ^c^*R*_free_ | 0.29 / 0.34 |
| No. reflections all / free | 10942 / 591 |
| No. Atoms Protein | 2690 |
| *B*-factors Protein (Å^2^) | 69.76 |
| R.m.s. deviations | |
| Bond lengths (Å) | 0.012 |
| Bond angles (°) | 1.72 |
| Ramachandran Plot (%) | |
| Favored Regions | 93.52 |
| Allowed Regions | 6.48 |

*Material and Methods: Detailed Prediction of epitopes: MLCE*

MLCE is a technique based on the analysis of the interaction energies of all the amino acids in a protein. In particular, it computes the non-bonded part of the potential (van der Waals, electrostatic interactions, solvent effects) via a MM/GBSA calculation, obtaining, for a protein composed by N residues, a N×N symmetric interaction matrix
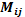

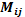
. This matrix can be expressed in terms of its eigenvalues and eigenvectors as


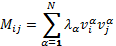


where
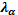

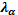
is the α-th eigenvalue and
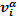

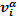
is the i-th component of the corresponding eigenvector. The eigenvector with the most negative correspondent eigenvalue contains most of the interaction information for the stabilizing interaction of the system. An approximated interaction matrix
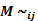

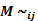
is thus given by


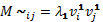


If the structure of the protein is known, one can estimate a contact matrix
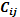

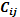
by assuming two amino acids in contact if the distance between two of their heavy atoms is smaller than a threshold. The Hadamard product of the two matrices gives us the matrix of the local coupling energies.


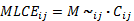


We select as possible interacting zones sets of close by residues that show weak or frustrated interactions.

The analysis of the energetic properties of the surface residues is based on the MLCE method. Basically, we perform a MM/GBSA analysis of the structure in a force field, obtaining a symmetric per-residue interaction matrix
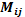

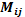
keeping only non-bonded interaction (i.e. electrostatic, van der Waals and solvation contributions). We diagonalize the matrix, obtaining a set of eigenvectors
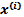

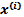
sorted following the increasing value of their eigenvectors
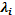

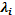
where N is the number of amino acids in the sequence. We thus can write the original matrix
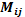

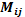
as


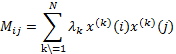


It has been shown that the first eigenvector alone can be used to build an approximate interaction matrix
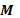

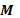
 :
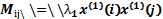

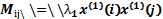
, which recapitulates the interactions most relevant for the stabilization of a certain conformation of a defined protein or protein substructure.

The MM/GBSA is performed with Amber 14 software using the ff14SB forcefield.


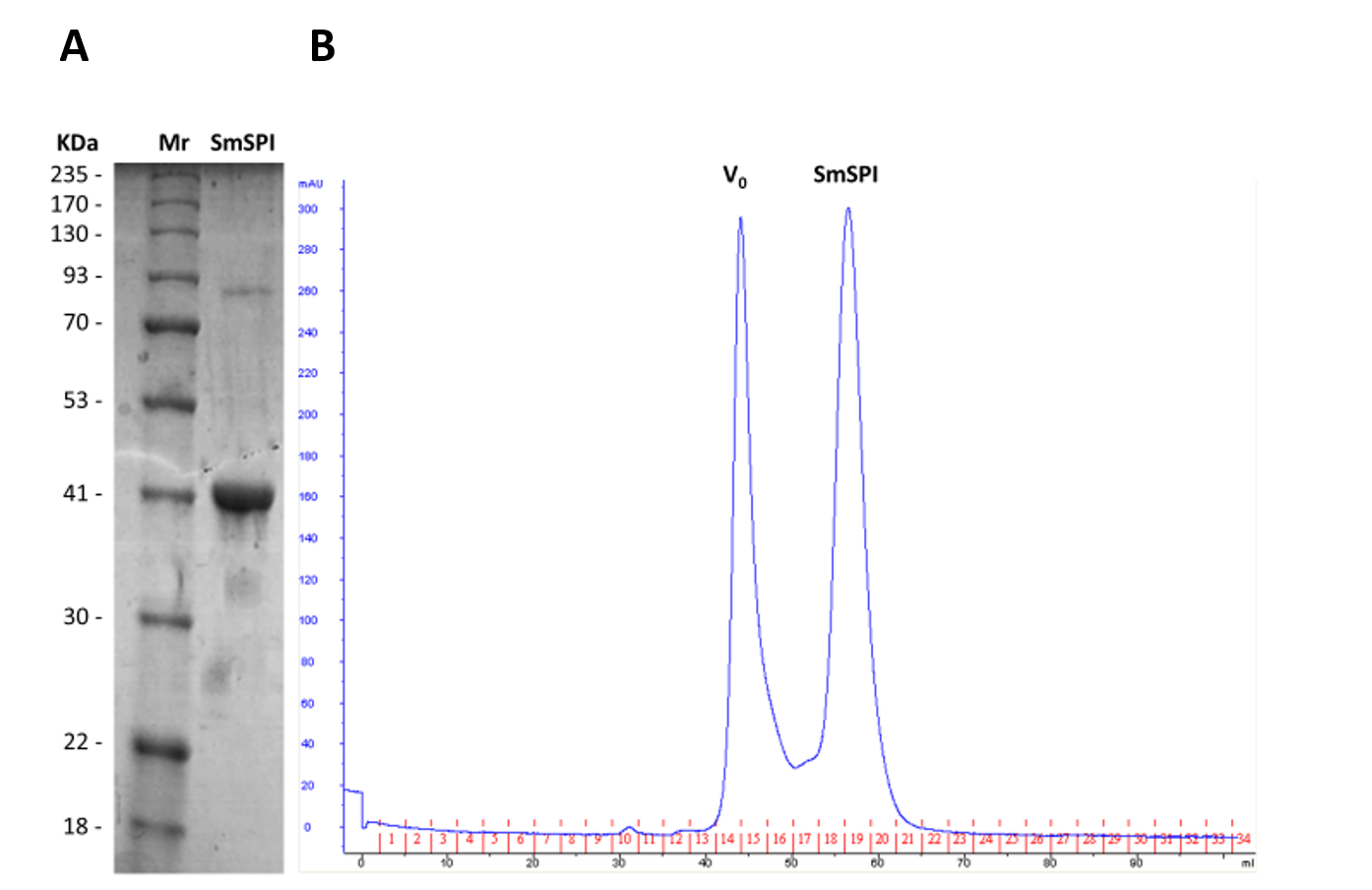


**Figure S1.** SDS-PAGE and gel filtration chromatogram. Panel A: SDS-PAGE in non-reducing condition of the purified SmSPI protein. The protein runs on a 12% Tris-glycine precast gel (Genscript) run in 1x MOPS with an approximate Molecular Weight (MW) of 44 kDa, in agreement with the calculated MW after the cleavage of the tag. Panel B: gel filtration chromatogram of SmSPI. The protein of interest elutes in a single sharp peak well resolved from its contaminants eluting in V_0_.

**Figure S2.** Alignment of the sequences used to generate ENDscript Sausage representation of the conserved regions among the SmSPI 153 structural homologs. 6SSV represents SmSPI sequence used as reference.

In silico epitope predictions


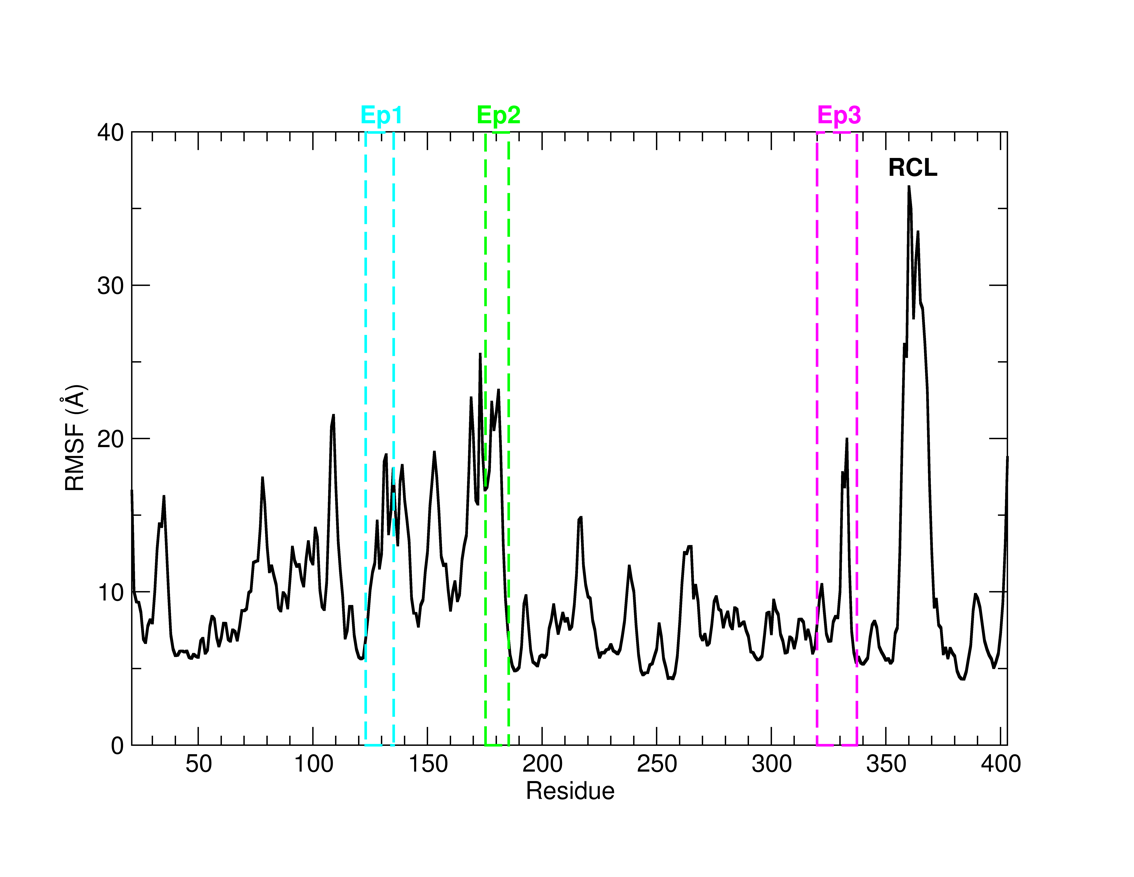


**Figure S3.** **Root Mean Square Fluctuation (RMSF) of SmSPI (3x1μs MD simulations).** The predicted epitopes are represented in cyan, green, and pink broken lines for Ep1, Ep2 and Ep3, respectively.
